# Origin of Asymmetric Electric Double Layers at Electrified Oxide/Electrolyte Interfaces

**DOI:** 10.1021/acs.jpclett.1c00775

**Published:** 2021-05-11

**Authors:** Mei Jia, Chao Zhang, Jun Cheng

**Affiliations:** † State Key Laboratory of Physical Chemistry of Solid Surfaces, iChEM, College of Chemistry and Chemical Engineering, Xiamen University, Xiamen 361005, China; ‡ Department of Chemistry-Ångström Laboratory, Uppsala University, Lägerhyddsvgen 1, P.O. Box 538, 75121 Uppsala, Sweden

## Abstract

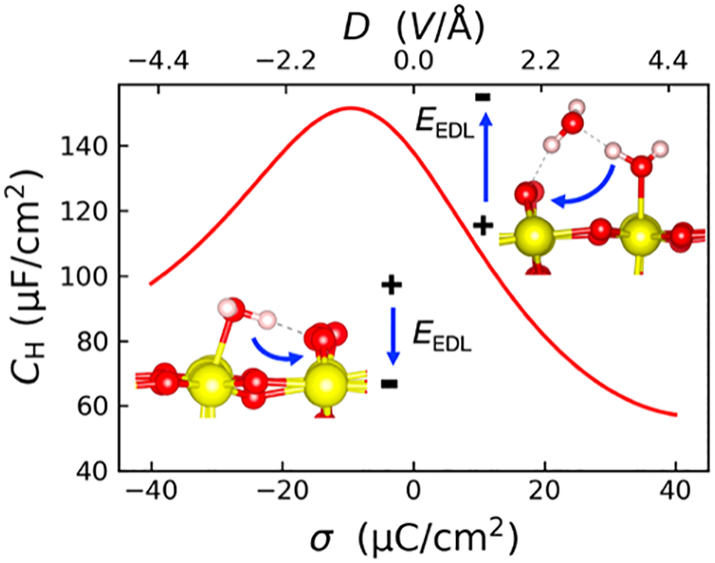

The structure of electric double layers (EDLs) dictates the chemistry and the physics of electrified interfaces, and the differential capacitance is the key property for characterizing EDLs. Here we develop a theoretical model for computing the differential Helmholtz capacitance *C*_H_ of oxide–electrolyte interfaces using density functional theory-based finite-field molecular dynamics simulations. It is found that the dipole of interfacial adsorbed groups (i.e., water molecule, hydroxyl ion, and proton) at the electrified SnO_2_(110)/NaCl interfaces significantly modulates the double layer potential which leads to the asymmetric distribution of *C*_H_. We also find that the dissociative water adsorption prefers the inner sphere binding of counterions, which in turn leads to a higher Helmholtz capacitance, compared with that of the nondissociative case at the interface. This work provides a molecular interpretation of asymmetric EDLs seen experimentally in a range of metal oxides/hydroxides.

Semiconducting oxide–electrolyte interfaces are highly electrified under working conditions.^1,2^ The charges of semiconducting oxide surfaces come from two sources. The difference in the electrochemical potentials between oxide and electrolyte leads to the depletion or accumulation of electrons between two phases and, therefore, the formation of the space charge layer. In addition, the adsorption of protons and hydroxyl groups generates positive charges and negative charges, respectively, at these oxide surfaces.^3^ The net interfacial proton charge is compensated by counterions from the electrolyte, which builds up the so-called protonic electric double layer (EDL),^4^ which spans for just 3–5 Å, that is, the Helmholtz layer, at high ionic strength and under the flat band potential condition.^4,5^

EDLs play an important role in electrochemistry,^6,7^ photoelectrocatalysis,^8^ colloid science, and geochemistry. Some of the fundamental questions therein are the following: What are the surface composition and ions distribution in the EDL? How would adsorbed water molecules respond to the electric field?^9^ How would the dissociation and the recombination of interfacial water molecules affect the double layer potential Δ*U*? What would the differential capacitance look like, that is, the change of the Helmholtz capacitance with respect to the surface charge density?^10^ In this regard, experimental methods such as X-ray photoelectron spectroscopy and X-ray standing wave provide the ion adsorption and the surface potential information;^11,12^ the sum-frequency generation spectroscopy probes the polarization of water at charged interfaces;^13,14^ and titration experiments reveal the change of surface charge density versus pH.^15,16^ Nevertheless, the analysis and extraction of this microscopic interfacial information on EDL is rather difficult if not impossible.^17−19^

This calls for density functional theory-based molecular dynamics simulation (DFTMD), which is a suitable computational method for describing the microscopic structure and the dynamics of water and ions near an interface.^20,21^ Recently, it has been shown that the finite-field DFTMD is a promising technique for modeling EDLs at electrified solid–liquid interfaces.^22−24^ Finite-field DFTMD simulations relies on the constant electric displacement *D* Hamiltonian introduced by Stengel, Spaldin, and Vanderbilt,^25^ and the corresponding expression of the average Helmholtz capacitance *C*_H_ is shown to be^22^
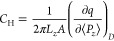
1where *L*_*z*_ is the dimension of the supercell perpendicular to the interface, *q* is the introduced surface proton charge, *A* is the area of the *x*, *y* cross section, and ⟨*P*_*z*_⟩ indicates the ensemble averaged supercell polarization. Because ⟨*P*_*z*_⟩ converges rapidly in the finite-field simulations, this makes *C*_H_ a new observable within the accessible time-scale of DFTMD. Note that in conjugation with eq 1, two sides of the oxide slab take opposite types but the same amount of proton charges in the simulated supercell.

In the first work of applying finite-field DFTMD for modeling EDLs,^23^ it was found that the Helmholtz capacitance at electrified rutile TiO_2_(110)/NaCl interfaces is much higher at high pH than that at low pH for the given surface charge density, and the interfacial proton transfer at low pH increases significantly the capacitance value. Compared with rutile TiO_2_, the isostructural cassiterite SnO_2_ has a characteristic dissociative water adsorption,^26,27^ which involves terminal adsorbed water Sn_5c_O_w_H_2_ to bridge oxygen site Sn_2_O_br_ as
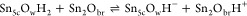
2

Then, the question naturally arises: how would the proton transfer affect the differential capacitance and vice versa? To answer that, herein the electrified SnO_2_(110)/NaCl electrolyte interfaces at different surface charge densities σ were simulated with finite-field DFTMD, using the CP2K/Quickstep package^28,29^ with the PBE functional.^30^ Detailed descriptions of the computational setup are given in the Supporting Information.

At low pH, the SnO_2_(110) surfaces are positively charged by adsorbing protons to Sn_2_O_br_ sites, and the direction of dissociative proton transfer is opposite to that of the electric field E in the EDL (Figure 1a, I and II). At high pH, the SnO_2_(110) surfaces are negatively charged by the desorption proton from terminal Sn_5c_O_w_H_2_, and the direction of the dissociative proton transfer is the same as that of the electric field E in the EDL (Figure 1a, III and IV). Because of this contrast, the degree of interfacial water dissociation (reaction 2) α increases with the pH in electrolyte (Figure 1b), when averaging the number of Sn_5c_O_w_H_2_, Sn_5c_O_w_H^–^, Sn_2_O_br_H^+^, and Sn_2_O_br_ sites (16 sites in total on each surface) over DFTMD trajectories (see Supporting Information). Meanwhile, we find the free energy of water dissociation (Reaction 2) Δ*A*_diss_ decreases with the pH (see Supporting Information).

**Figure 1:**
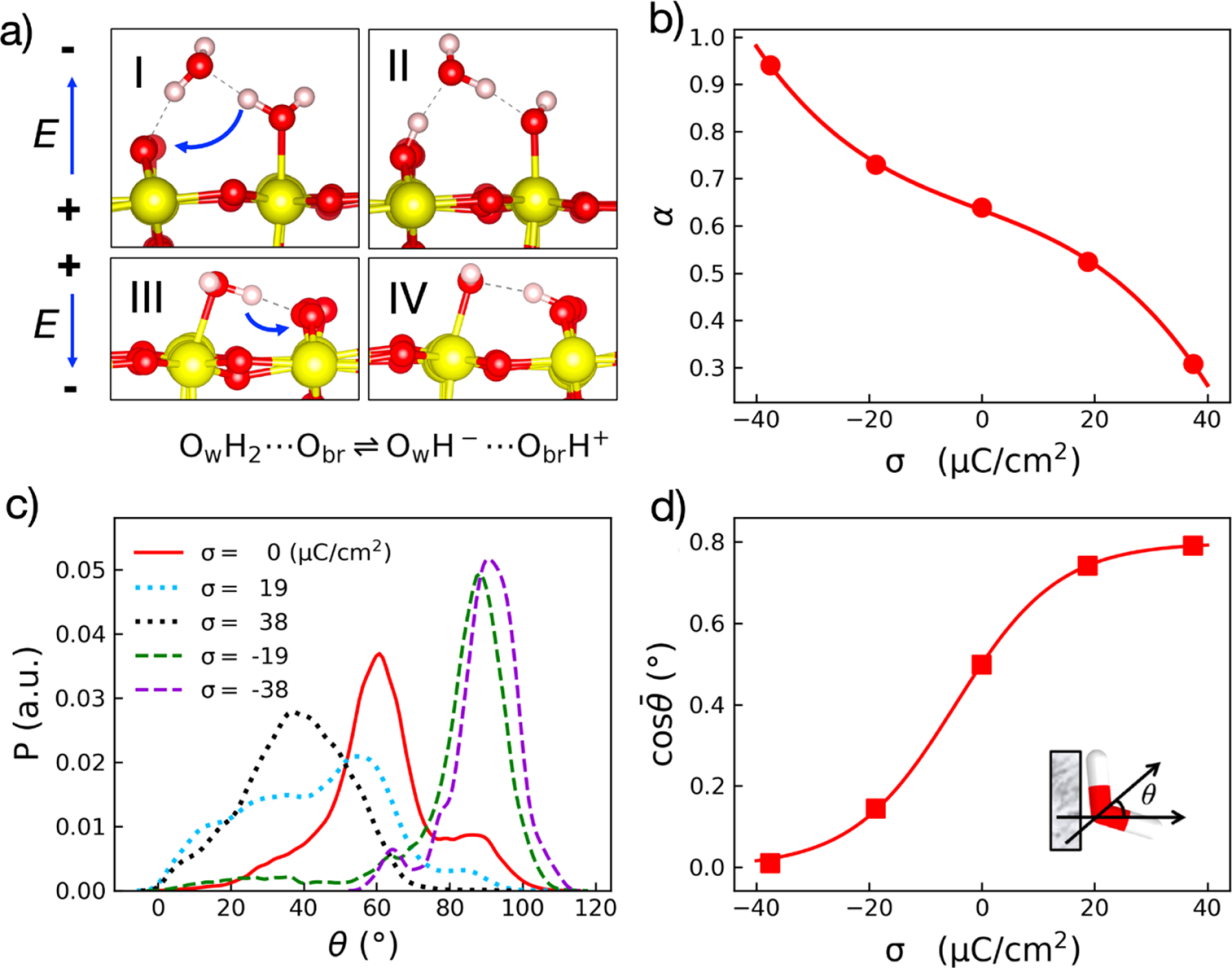
(a) Dissociation of interfacial water molecules at the low pH (I and II) and high pH (III and IV) interfaces in response to the electric field E. The blue arrows on the left side show the direction of the electric field E of the double layer. (b) Plot of the degree of water dissociation α as a function of surface charge density σ. (c) Probability distribution of the angle θ between the bisector of adsorbed water molecules and surface normal for SnO_2_(110)/NaCl interfaces at different σ. (d) Plot of cos θ̅ of adsorbed water molecules as a function of σ.

When looking at the orientation of interfacial water, it is found that the adsorbed water molecules point toward the electrolyte solution (Figure 1c), with the angle θ between the water dipole and surface normal of about 60° at the PZC. This value is down-shifted to 30°–40° and up-shifted to 80°–90°, in response to the electric field in EDL. As shown in Figure 1d, the average angle of adsorbed water molecules shows a monotonic increment as a function of σ in spite of a strong presence of the water dissociation.

It has long been known that the dipole of interfacial hydroxyl groups at oxide–electrolyte interfaces strongly affect the potential offset.^31−33^ As shown in Figure 2a (see Supporting Information), the band edge is shifted upward with reference to the standard hydrogen electrode (SHE) scale^34^ when going from the vacuum surface, to the water monolayer (ML) adsorption and the fully solvated SnO_2_(110)/H_2_O interface. This effect is also manifested when restraining water molecules adsorbed on the left side of the SnO_2_ slab not undergoing dissociation reactions at the PZC. In this case, interfacial water molecules would have different orientational distributions on the two sides of the SnO_2_ slab (Figure 2b), and the average θ of the left side (restrained) is about 10° larger than that of the right side (free). This leads to a total net dipole pointing to the opposite of the surface normal (Figure 2c). As a consequence and illustrated in Figure 2d, the left side (restrained) of the slab has a higher potential than that of the right side.

**Figure 2:**
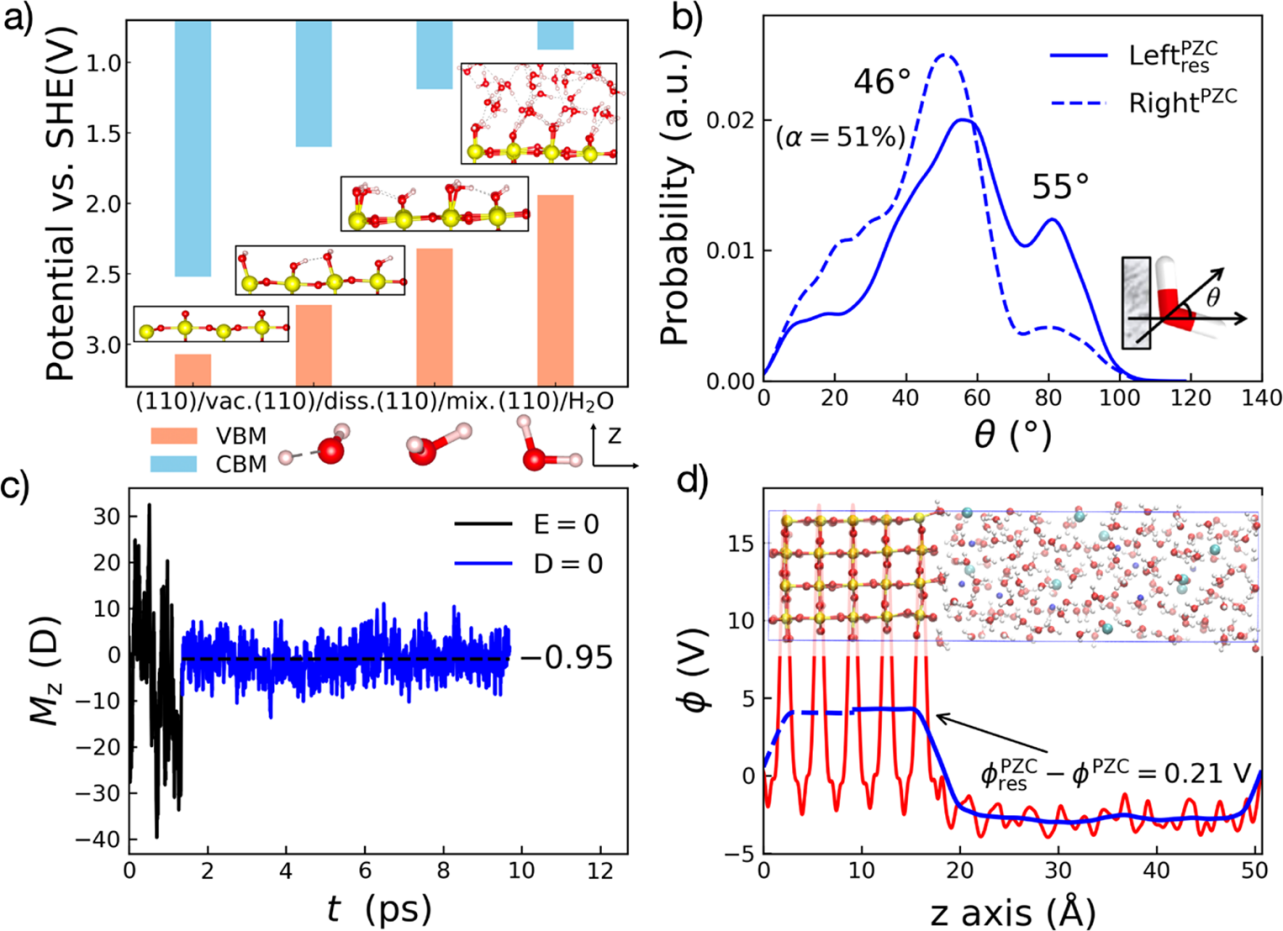
(a) Band alignment of SnO_2_(110)/vacuum interface ((110)/vac.), a monolayer of dissociatively adsorbed water interfaces ((110)/diss.), mixed molecularly adsorbed and dissociatively adsorbed water interface ((110)/mix.), and the SnO_2_(110)/H_2_O interface ((110)/H_2_O) from left to right. The insets are the corresponding simulated models. (b) Probability distribution of the angle θ between the bisector of adsorbed water molecules and surface normal for the left side restrained (Left_res_^PZC^) and the right side of SnO_2_(110)/NaCl interface (Right^PZC^) at PZC. “Restrained” means the O–H bond of adsorbed water molecules were attached with a restraining potential to prevent the dissociation (see Computational setup). (c) Time evolution of the total dipole moment *M*_*z*_ for left side restrained SnO_2_(110)/NaCl interface at PZC, when switching the electric boundary condition. Black dashed line is the time average of *M*_*z*_. (d) The electrostatic potential ϕ profile at PZC. ϕ_res_^PZC^ and ϕ^PZC^ are electrostatic potentials for left restrained side and right side.

On the basis of these observations made in Figure 1 and 2, we have formulated a theoretical model of differential Helmholtz capacitance at oxide-electrolyte interfaces to take the water dipole contribution into account explicitly. We begin with the textbook definition of the capacitance *C*_H_
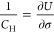
3where *U* is the potential drop across the Helmholtz layer, and σ is the surface charge density.

Then, applying the fundamental relation *D* = *E* + 4*πP* and using the field expressions instead of potential and charge density, we can rewrite the above expression as 
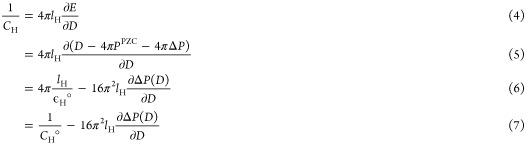
where *l*_H_ is the width of the Helmholtz layer, ϵ_H_° is the corresponding interfacial dielectric constant. *P*^PZC^ is the total polarization at the PZC, and Δ*P* = *P* – *P*^PZC^, which contains the difference in the polarization with respect to PZC.

From the equation above, it is clear that we will have a constant term *C*_H_°, which is independent of *D*, and a water polarization term Δ*P*(*D*), which is a function of *D*.

In case we are using the polarization of adsorbed surface O–H groups Δ*P*_w_ as a descriptor, the Δ*P*_w_ includes the contribution of adsorbed water molecules in both molecular and dissociative forms. One may scale Δ*P*_w_ by the dielectric constant of the double layer ϵ_H_° to account for the electrostatic screening. This recasts eq 7 into the following form:
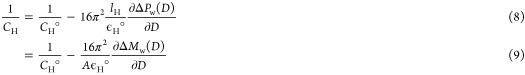
where Δ*M*_w_(*D*) = Ω · Δ*P*_w_(*D*), the volume Ω = *A* · *l*_H_. Here Δ*M*_w_(*D*) is the total dipole moment difference of interfacial groups (including the molecular and dissociative forms of adsorbed water) with respect to PZC. We can get Δ*M*_w_ = (*M*_w_ · *N*_w_ – *M*_w_^0^ · *N*_w_^0^) + (*M*_dis_ · *N*_dis_ – *M*_dis_^0^ · *N*_dis_^0^). *M*_w_, *M*_dis_, *N*_w_, and *N*_dis_ are the dipole moment and number of adsorbed water molecule and dissociated (OH^–^+H^+^) groups (see Figure S5a). Note that in the model introduced here, there is only one free parameter ϵ_H_° in eq 9. Instead, both *l*_H_ and Δ*M*_w_(*D*) can be determined from finite-field DFTMD simulations. This allows us to obtain the differential capacitance rather than applying the one-shot estimator (eq 1).

Then, we applied the eq 9 to fit *U* – *U*^PZC^, that is, the potential drop crossing the Helmholtz layer with respect to that at the PZC, which can be obtained from finite-field DFTMD trajectories using the macroscopic averaging technique^35^ (see Figure 3a and Figure S6a). In the case of SnO_2_(110), Δ*M*_w_(*D*) includes the contribution of adsorbed water molecules in both molecular and dissociative forms as shown in Figure 3b,d. The width of the Helmholtz layer *l*_H_ is about 2.6 Å, which estimates from the surface normal projected distance between the counterions and the nuclei of O_br_ sites averaged over all surface charge densities.

**Figure 3:**
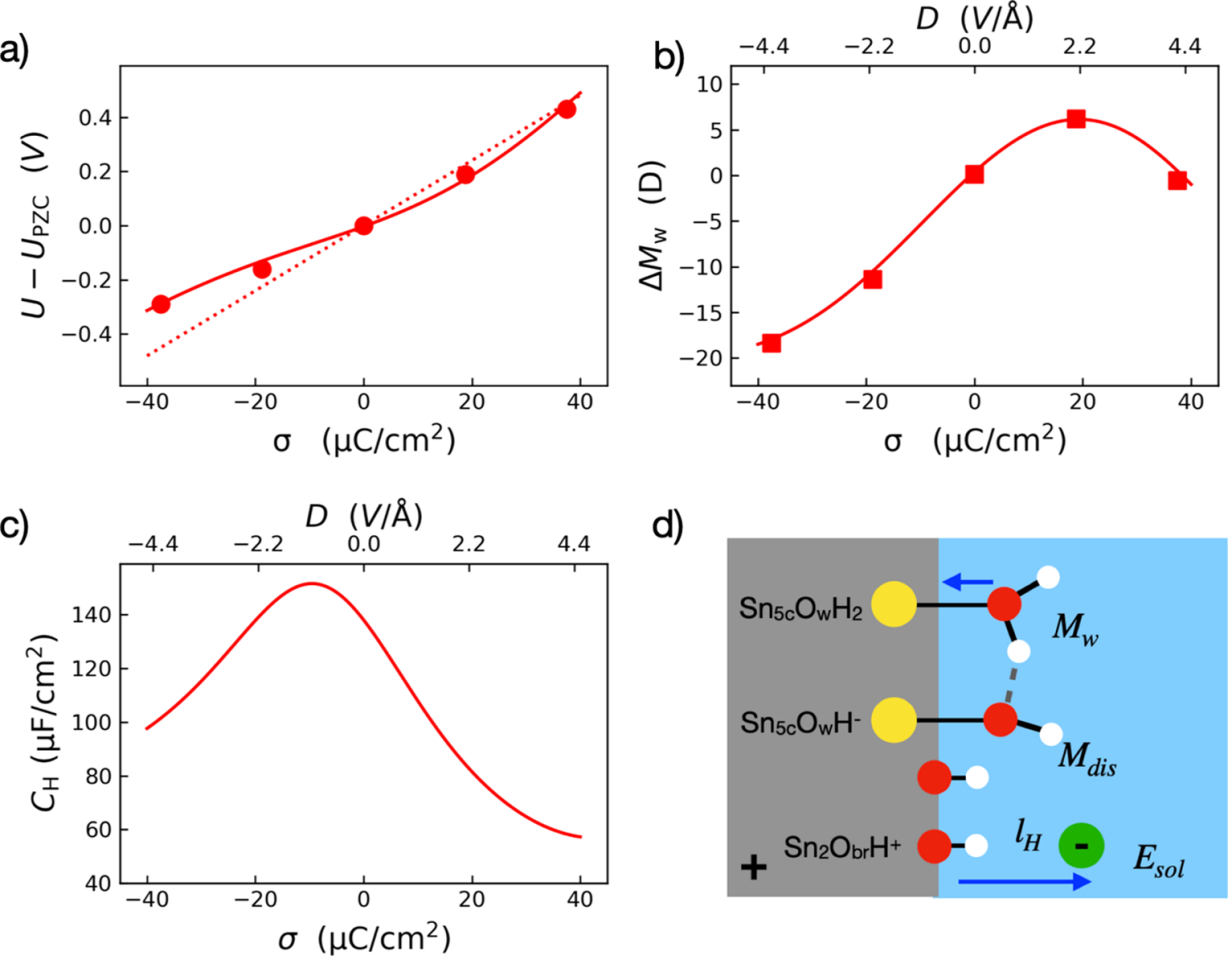
(a) Potential drop across the Helmholtz layer with respect to that at PZC. (b) Variation of the total dipole moment difference of interfacial groups (including the molecular and dissociative forms of adsorbed water) Δ*M*_w_(*D*) with respect to PZC. (c) The differential capacitance of the Helmholtz layer *C*_H_ as a function of surface charge density σ. (d) A schematic illustration of the coupling between the EDL field and the water orientation/dissociation at the SnO_2_(110)/NaCl interface at low pH.

The fitting result of *U*(σ) – *U*^PZC^ is given in Figure 3a, where the dashed line indicates the first constant term in eq 9. The free parameter ϵ_H_° turns out to be 24, which is quite close to the commonly assumed values for rutile structures.^36^ This agreement further justifies the theoretical model we formulated for the differential Helmholtz capacitance at oxide–electrolyte interfaces.

By taking the analytical derivative of *U*(σ) – *U*^PZC^, we obtain the differential capacitance *C*_H_ of the Helmholtz layer as shown in Figure 3c. We find that *C*_H_ shows a maximum of ∼151 μF/cm^2^ at the negatively surface charge density −10 μC/cm^2^, and it decreases to ∼100 and ∼60 μF/cm^2^ when the surface charge density moves away to −40 and 40 μC/cm^2^, respectively. In addition, we find that *C*_H_ at the negatively charged surface is about 50% higher than that at the positively charged surface for the same value of |σ|, and this finding of asymmetric distribution of the differential *C*_H_ is in accord with what has been seen at electrified TiO_2_(110)/NaCl interfaces from finite-field DFTMD simulations^23^ and in agreement with titration experiments of SnO_2_ at higher ionic strength.^15,37^ In fact, the asymmetric Helmholtz capacitance has been also seen in a range of metal oxides/hydroxides, such as ZnO,^38^ TiO_2_,^16^ α-Al_2_O_3_^39^ and γ-FeOOH.^40^

This common feature observed in different oxides suggests that there should be a fundamental reason behind its cause. Because the model given in eq 9 successfully captures the variation of *U*(σ) – *U*^PZC^ with Δ*M*_w_ as the input, this points to the asymmetric orientation of adsorbed surface O–H groups as the determining factor. At the metal oxide surfaces, water molecules have preferred orientations because of the chemisorption, where dipoles are pointing to the liquid phase, i.e. ⟨*M*_w_⟩ > 0 at PZC (Figure 1c). This surface effect can be captured by introducing an auxiliary field *D*^PZC^ into the Debye-type Hamiltonian – μ · (*D* + *D*^PZC^) cos(θ), where μ is the dipole moment of noninteracting water molecules.

Then, one can show that the ensemble averaged Δ⟨*M*_w_⟩ with reference to the PZC (Figure 4a) is
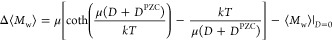
10When taking the derivative of eq 10 with respect to *D* and combining it with eq 7, this leads to the expression of the differential capacitance *C*_H_ at the oxide surface (Figure 4b) as
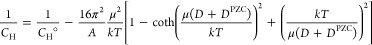
11It is worth stressing that *D*^PZC^ is not due to the proton charge as in *D* but an intrinsic property of metal oxide surfaces. Moreover, *D*^PZC^ is positive, as in the same direction of the water dipole at the PZC (Figure 1c). This offset *D*^PZC^ due to the specific orientation of adsorbed water at the PZC is the origin for the asymmetric electric doubles layers at oxide-electrolyte interfaces.

**Figure 4:**
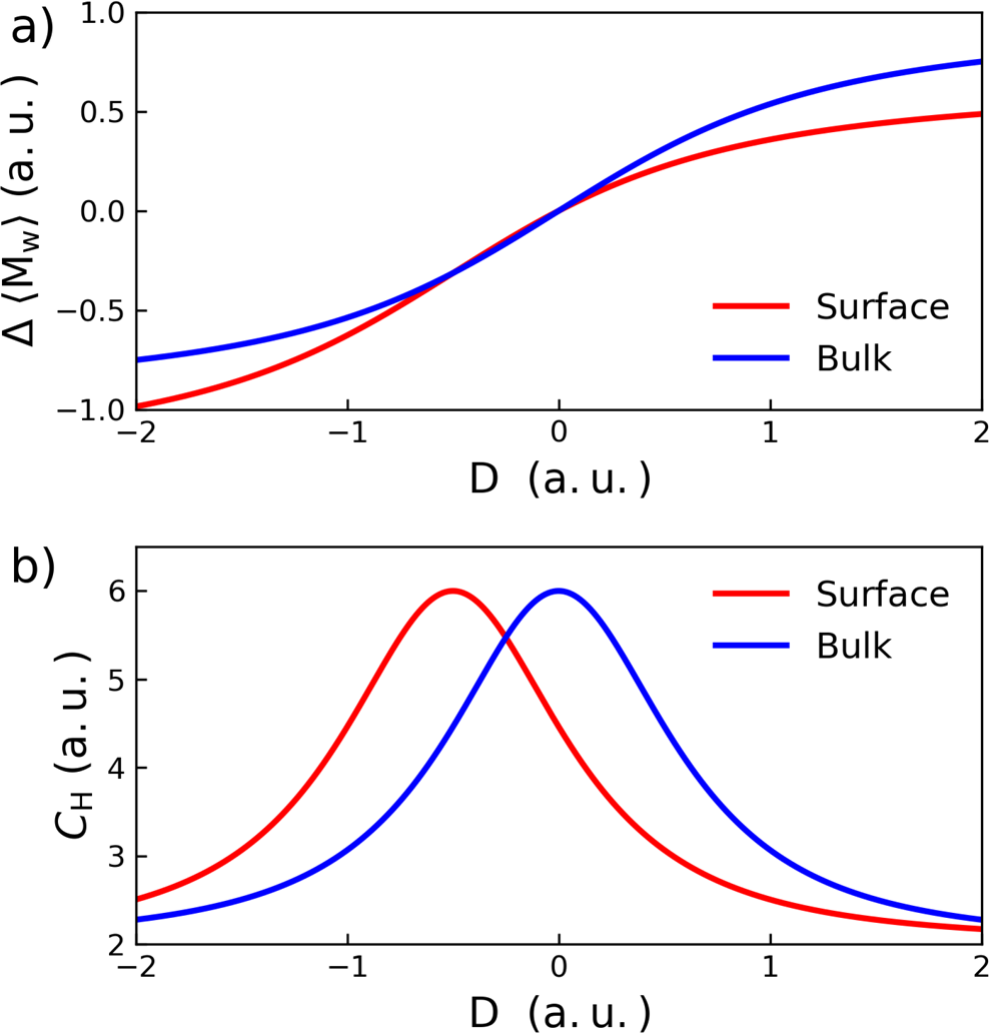
(a) Dependence of the dipole Δ⟨*M*_w_⟩ as a function of the electric displacement *D* according to eq 10. (b) The differential capacitance profile as a function of the electric displacement *D* according to eq 11.

The final question is how the chemical specificity of surfaces comes into play. Clearly, the magnitude and the sign of *D*^PZC^ is system-dependent. More importantly, in the case of SnO_2_(110), the interaction between dissociated OH^–^, H^+^ and counterions in electrolyte is direct and strong, forming the inner sphere coordination. For example, at the positively charged interfaces, as shown in Figure 5a,e, the counterion Cl^–^ is stabilized by two neighboring Sn_2_O_br_H^+^ and two Sn_5c_O_w_H^–^ groups. Similarly, at the negatively charged interfaces, the counterion Na^+^ is stabilized by two neighboring Sn_5c_O_w_H^–^ or two Sn_2_O_br_ groups as shown in Figure 5b,f.

**Figure 5:**
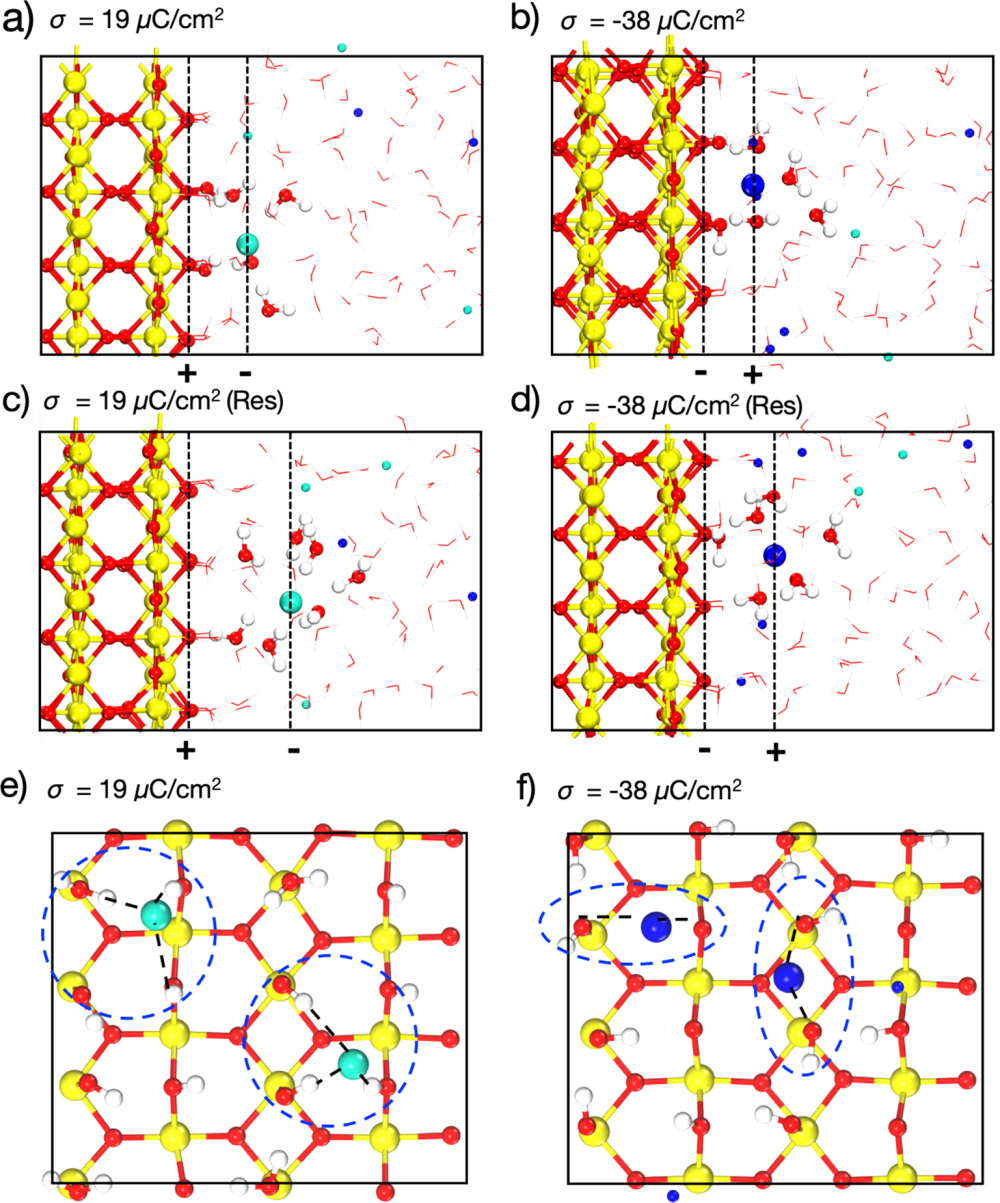
Side view (a–d) and top view (e,f) of the electrified SnO_2_(110)/NaCl electrolyte interface models at σ = 19 and −38 μC/cm^2^. Note that the adsorbed water molecules of (c) and (d) were restrained and not undergoing the dissociation. The Sn, O, H, Na, and Cl atoms were colored in yellow, red, white, blue, and cyan, correspondingly. The water molecules and surface O–H groups coordinated to counterions were highlighted with the ball–stick model.

In contrast, the outer sphere coordination is preferred when restraining adsorbed water molecules not undergoing the dissociation (Figure 5c,d). Consequently, the average *C*_H_ of SnO_2_ with dissociatively adsorbed water molecules is about 109 μF/cm^2^, which is 45% larger than that of SnO_2_ with water molecules restrained to the molecular adsorption ∼61 μF/cm^2^ (Figure S7c). Overall, they indicate that theoretical models connecting the macroscopic property and the microscopy information are essential for interpreting the differential capacitance *C*_H_ of oxide–electrolyte interfaces.

In summary, through a combination of finite–finite DFTMD simulations and a theoretical model, the quantitative relationship between interfacial water orientation, proton transfer and differential capacitance at the charged water interface of SnO_2_(110) was established. It has been shown that the general phenomenon of asymmetric electric double layers seen in a range of metal oxides/hydroxides can be explained by the specific orientation of chemisorbed water molecules at the point of zero charge.

## Supporting Information Available

Detailed descriptions of computational setup, the degree of water dissociation α, the free energy of water dissociation Δ*A*_diss_, band edge alignment, double layer potentials and differential capacitance at electrified SnO_2_(110)/NaCl interfaces (PDF)
