# Supplementary material for: Origin of Asymmetric Electric Double Layers at Electrified Oxide/Electrolyte Interfaces
Source: J Phys Chem Lett. 2021 May 11;12(19):4616–22. doi: 10.1021/acs.jpclett.1c00775 (PMC8154876; doi:10.1021/acs.jpclett.1c00775)
Supplement: Supplementary file 1 — jz1c00775_si_001.pdf [file jz1c00775_si_001.pdf]

# Supporting Information for Origin of asymmetric electric double layers at electrified oxide/electrolyte interfaces

Mei Jia,[†] Chao Zhang,[*,‡] and Jun Cheng[*,†]

†*State Key Laboratory of Physical Chemistry of Solid Surfaces, iChEM, College of Chemistry and Chemical Engineering, Xiamen University, Xiamen 361005, China*

‡*Department of Chemistry-Ångström Laboratory, Uppsala University, Lägerhyddsvägen 1, P. O. Box 538, 75121 Uppsala, Sweden*

E-mail: chao.zhang@kemi.uu.se; chengjun@xmu.edu.cn

## Contents

**1 Computational setup of electrified $SnO_2(110)$/NaCl electrolyte interfaces S2**

**2 The degree of water dissociation $\alpha$ S3**

**3 The free energy of water dissociation $\Delta A_{\mathrm{diss}}$ S4**

**4 Band edge alignment at $SnO_2(110)$ surfaces S4**

**5 Double layer potentials at $SnO_2(110)$/NaCl interfaces S5**

**6 Differential capacitance at restrained $SnO_2(110)$/NaCl interfaces S5**

**References S14**

# 1 Computational setup of electrified $SnO_2(110)$/NaCl electrolyte interfaces

The $SnO_2(110)$ surface was modeled by a symmetric periodic slabs of five O-Sn-O layers with lateral dimensions of a 4 × 2 surface cell. The slabs were separated by an electrolyte space of 34.6 Å giving an orthorhombic supercell of dimensions 12.7 × 13.4 × 50.6 Å$^3$. A molality of 2.6-2.7 mol/kg (the concentration of the electrolyte used in industry) for NaCl electrolyte was simulated in our systems. The space between the $SnO_2$ slab was filled with 186 water molecules, 9 $Na^+$ and 9 $Cl^-$ in each unit cell (see Supporting Information Figure S1 and S2). This ensure the right bulk density as shown in Supporting Information Figure S3.

For all the cases of electrified $SnO_2(110)$/NaCl electrolyte interfaces calculations, classical MD simulations were performed with GROMACS for the pre-equilibration of the system[1,2] before applying the hybrid SSV electric displacement constant $\bar{D}$ based DFTMD simulations.[3–5] The detail of finite field methods for the supercell modeling of charged insulator/electrolyte interfaces is described in Secs. IV in Ref.[5]

The PBE functional[6] was applied to all the simulations of unrestrained and restrained electrified $SnO_2(110)$/NaCl electrolyte interfaces. Goedecker-Teter-Hutter (GTH) pseudopotentials[7,8] were employed to represent the core electrons. The atomic basis sets for the valence electrons were the standard short-ranged double-$\zeta$ basis functions with one set of polarization functions (DZVP).[9] The plane wave cutoff for electron density expansion was set as 400 Ry. The target accuracy for the SCF convergence was 3 × $10^{-7}$ a.u.. The NVT ensemble was used for MD propagation with the time step of 0.5 fs and the equilibrium temperature for all the simulations was kept to be 330K by using the Nosé-Hoover thermostat.[10] All the DFTMD calculations were carried out by using the CP2K/Quickstep package.[11,12] 2 $\sim$ 4 ps of initial equilibration at $\bar{E}$= 0 condition was followed by 8 $\sim$ 30 ps of production period at $\bar{D}$ = 0 condition for each step-up (Supporting Information Figure S4).

For the "restrained" systems in this work, the O-H bonds of interfacial groups ($Sn_{5c}O_wH_2$, $Sn_{5c}O_wH^-$ and $Sn_2O_{br}H^+$) were attached with a harmonic restraining potential $V_r$ to prevent the dissociation. The form of the harmonic restraining potential $V_r$ is written as[13]

$$V_r = \sum_{bonds} \frac{k_r}{2}(r - r_{eq})^2 \tag{S1}$$

where $k_r$ and $r_{eq}$ are the force constant and equilibrium value for the O-H bond lengths $r$. Here the two parameters $k_r$=0.2 and $r_{eq}$=1.89 Bohr were used in our simulations.

# 2 The degree of water dissociation $\alpha$

There are 8 $Sn_{5c}$ sites and 8 $Sn_2O_{br}$ sites in total on each surface. Water molecules adsorbed on terminal $Sn_{5c}$ sites, and the degree of water dissociation (Reaction 2 in main text) depend on the number of surface groups with interfacial proton exchange. Here we counted the number of surface groups by setting the O-H bond length cutoff as 1.23 Å. The values $\alpha$ of at different surface charge densities $\sigma$ are calculated as

$$\alpha = \frac{\mathrm{n}(Sn_{5c}O_wH^-)}{8 - \mathrm{n}(Sn_{5c}O_wH_2)} \tag{S2}$$

The results are listed in the last column of Table S1.

Table S1: Average numbers of surface sites $Sn_{5c}O_wH_2$, $Sn_{5c}O_wH^-$, $Sn_2O_{br}H^+$ and $Sn_2O_{br}$ at different surface charge densities $\sigma$ with the O-H bond length cutoff set as 1.23 Å.

| $\sigma(\mu C/cm^2)$ | $Sn_{5c}O_wH_2$ | $Sn_{5c}O_wH^-$ | $Sn_2O_{br}H^+$ | $Sn_2O_{br}$ | $\alpha$ |
|---|---|---|---|---|---|
| $PZC_{avg}$[a] | 2.89 | 5.11 | 5.04 | 2.96 | 63.9% |
| 19 | 3.81 | 4.19 | 5.81 | 2.19 | 52.4% |
| -19 | 1.60 | 6.40 | 4.38 | 3.62 | 73.3% |
| 38 | 4.15 | 1.85 | 5.76 | 2.24 | 30.8% |
| -38 | 0.24 | 7.76 | 3.76 | 4.24 | 94.0% |

[a] $PZC_{avg}$ denotes that the numbers of surface sites at PZC are calculated from the average results of left and right interface.

## 3 The free energy of water dissociation $\Delta A_{\text{diss}}$

The free energy of water dissociation $\Delta A_{\text{diss}}$ for Reaction (2) in the Main Text is:

$$\Delta A_{\text{diss}} = -k_{\text{B}}T\text{In}\frac{[\text{Sn}_{5\text{c}}\text{O}_\text{w}\text{H}^-]\cdot[\text{Sn}_2\text{O}_{\text{br}}\text{H}^+]}{[\text{Sn}_2\text{O}_{\text{br}}]\cdot[\text{Sn}_{5\text{c}}\text{O}_\text{w}\text{H}_2]} \tag{S3}$$

With the number of $Sn_{5c}O_wH_2$, $Sn_{5c}O_wH^-$, $Sn_2O_{br}H^+$ and $Sn_2O_{br}$ sites listed in Table S1, we get the plot of $\Delta A_{\text{diss}}$ which decreases with the pH in electrolyte as shown in Fig. S5b. The dipole moment of adsorbed water molecule, dissociated ($OH^-$+$H^+$) and their difference are described as $M_w$, $M_{dis}$ and $M_w$-$M_{dis}$ as shown in Fig. S5a.

## 4 Band edge alignment at $SnO_2$(110) surfaces

To further investigate the energy level shift caused by the interfacial adsorbed groups at $SnO_2$(110) surfaces, the positions of valence band maximum(VBM) and conduction band minimum (CBM) were calculated (Table S2). Here we used the average energy of the highest occupied molecular orbital (HOMO) of the solid which could be indicated by $\varepsilon_{\text{HOMO}}$ to estimate the position of VBM, the average energy of the lowest unoccupied molecular orbital ($\varepsilon_{\text{LUMO}}$) to estimate the position of CBM. Note that the band edge aligning $\varepsilon_{\text{HOMO}}$ and $\varepsilon_{\text{LUMO}}$ to the standard hydrogen electrode (SHE) were calculated by referencing to the vacuum level, and then subtracting the absolute SHE potential of 4.44 V. Final expressions of VBM and CBM reference to SHE scale are

$$e_0U_{\text{VBM}}(\text{SHE}) = -\varepsilon_{\text{HOMO}} - e\phi_{\text{wat}}^{(\text{SnO}_2)} + \Delta_{\text{dp}}\text{A}_{\text{H}_3\text{O}^+}^{(\text{w})} - \Delta_{\text{f}}\text{G}_{\text{H}^+}^{\text{g,o}} - \Delta_{\text{zp}}\text{E}_{\text{H}^+(\text{OH}_2)} \tag{S4}$$

$$e_0U_{\text{CBM}}(\text{SHE}) = -\varepsilon_{\text{LUMO}} - e\phi_{\text{wat}}^{(\text{SnO}_2)} + \Delta_{\text{dp}}\text{A}_{\text{H}_3\text{O}^+}^{(\text{w})} - \Delta_{\text{f}}\text{G}_{\text{H}^+}^{\text{g,o}} - \Delta_{\text{zp}}\text{E}_{\text{H}^+(\text{OH}_2)} \tag{S5}$$

where $\phi_{\text{wat}}^{(\text{SnO}_2)}$ is the average electrostatic potential of $SnO_2$(110)/water interface, $\Delta_{\text{dp}}A_{\text{H}_3\text{O}^+}$ is the deprotonation free energy of $H_3O^+$(aq), $\Delta_f G_{\text{H}^+}^{g,o}$ corresponds to the formation free

energy of the gas-phase proton from a $\frac{1}{2}H_2$ molecule, where the state of $H^+$ in the gas phase corresponds to 1 mol· dm$^{-3}$, $\Delta_{zp}E_{H^+(OH_2)}$ is a correction for the zero-point energy of the O−H bond in $H_3O^+$(aq). The latter three terms are equal to 15.35 eV, 15.81 eV and 0.35 eV.

Table S2: Variation of the band edge alignment (vs. SHE) and adsorption energy (eV per molecule) of a monolayer (ML) of water molecules adsorbed on five layer $SnO_2$(110)/vacuum interface[a] and the $SnO_2$(110)/$H_2O$ interface

| | VBM (V) | CBM (V) | Band gap (V) | Adsorption energy (eV) |
|---|---|---|---|---|
| $SnO_2$(110)/Vacuum | 3.07 | 2.52 | 0.55 | |
| $SnO_2$(110)/diss. | 2.72 | 1.6 | 1.11 | -1.5 |
| $SnO_2$(110)/mix. | 2.32 | 1.19 | 1.13 | -1.5 |
| $SnO_2$(110)/$H_2O$ | 1.94 | 0.91 | 1.03 | |

[a] diss. means fully dissociative adsorption, and mix. is a mixed state with half water associatively adsorbed and half water dissociatively adsorbed

# 5 Double layer potentials at $SnO_2$(110)/NaCl interfaces

The potential drop crossing EDLs with respect to that at the PZC for both unrestrained and restrained systems, as obtained from the macroscopic averaging technique, is shown in Fig. S6.

# 6 Differential capacitance at restrained $SnO_2$(110)/NaCl interfaces

The main results for restrained $SnO_2$(110)/NaCl interfaces are shown in Fig. S7. The feature of asymmetric double layers remains the same and the the corresponding differential capacitance profile can be found in Fig. S7c.

Note that compared to the unrestrained system shown in the Main Text, the Helmholtz capacitance of the restrained system becomes much smaller. This agrees with the results using Eq.1 in the main text, as shown in Table S3.

Table S3: Helmholtz capacitances at low pH ($C_{\mathrm{H}}^{+}$) and at high pH ($C_{\mathrm{H}}^{-}$), the average Helmholtz capacitance $C_{\mathrm{H}}$ of both unrestrained and restrained $SnO_2(110)/NaCl$ interfacess at different surface charge densities $\sigma$.

| $\sigma(\mu\mathrm{C/cm^2})$ | $C_{\mathrm{H}}^{+}$ ($\mu\mathrm{F/cm^2}$) | $C_{\mathrm{H}}^{-}$ ($\mu\mathrm{F/cm^2}$) | $C_{\mathrm{H}}$ ($\mu\mathrm{F/cm^2}$) |
|---|---|---|---|
| 19 | 84 | 140 | 93 |
| 38 | 58 | 101 | 106 |
| 19(Res) | 48 | 79 | 51 |
| 38(Res) | 33 | 64 | 68 |

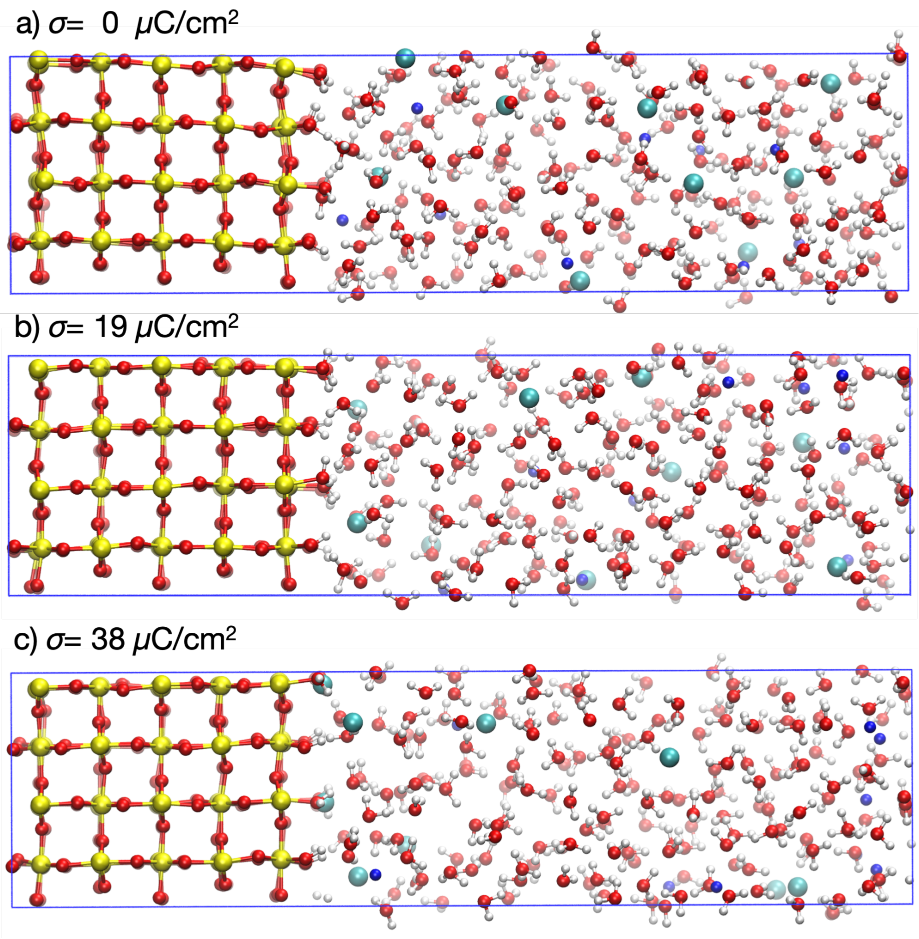


Figure S1: **Simulated models of the electrified $SnO_2$(110)/NaCl electrolyte interfaces.** Snapshots of the electrified $SnO_2$(110)/NaCl electrolyte interface models at surface charge density $\sigma = 0$ (a), $\sigma = 19$ (b) and $\sigma = 38$ (c) $\mu C/cm^2$. The Sn, O, H, Na and Cl atoms are colored by yellow, red, white, blue and cyan, respectively.

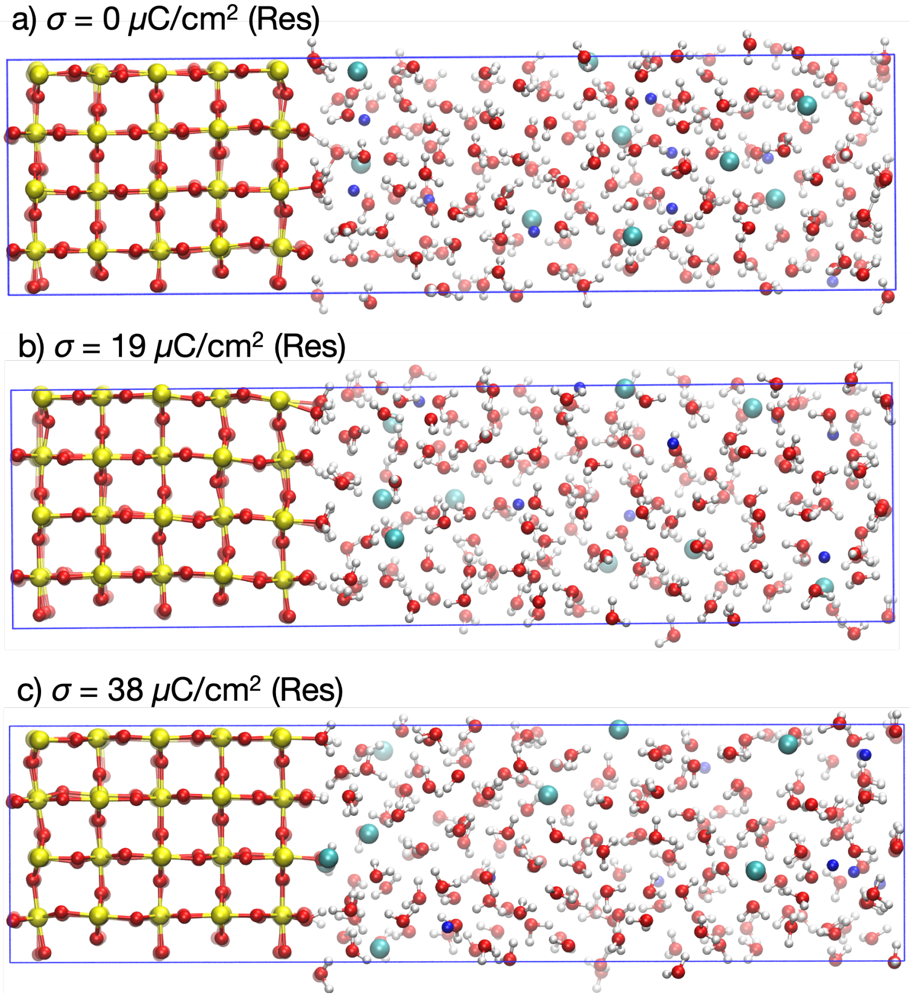


Figure S2: **Simulated restrained models of the electrified $SnO_2$(110)/NaCl electrolyte interfaces.** Snapshots of the restrained electrified $SnO_2$(110)/NaCl electrolyte interface models at surface charge density $\sigma = 0$ (a), $\sigma = 19$ (b) and $\sigma = 38$ (c) $\mu$C/cm$^2$. The Sn, O, H, Na and Cl atoms are colored by yellow, red, white, blue and cyan, respectively.

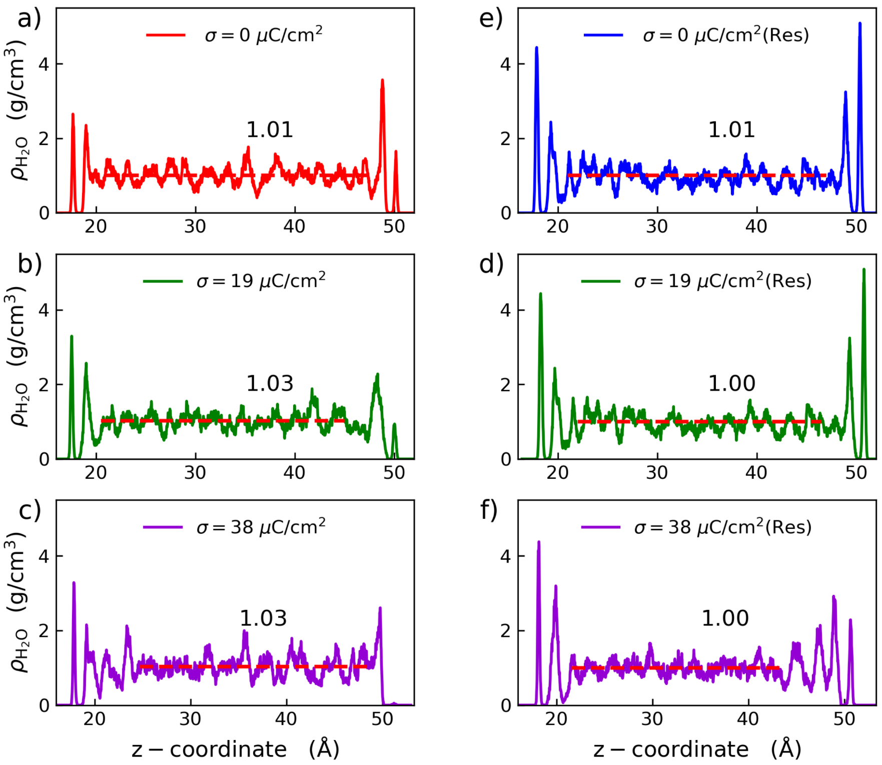


Figure S3: **Plots of water density at different surface charge densities.** The water density profiles of unrestrained (a, b, c) and restrained (d, e, f) electrified $SnO_2(110)$/NaCl electrolyte interface models at $\sigma$ = 0, 19 and 38 $\mu$C/cm$^2$.

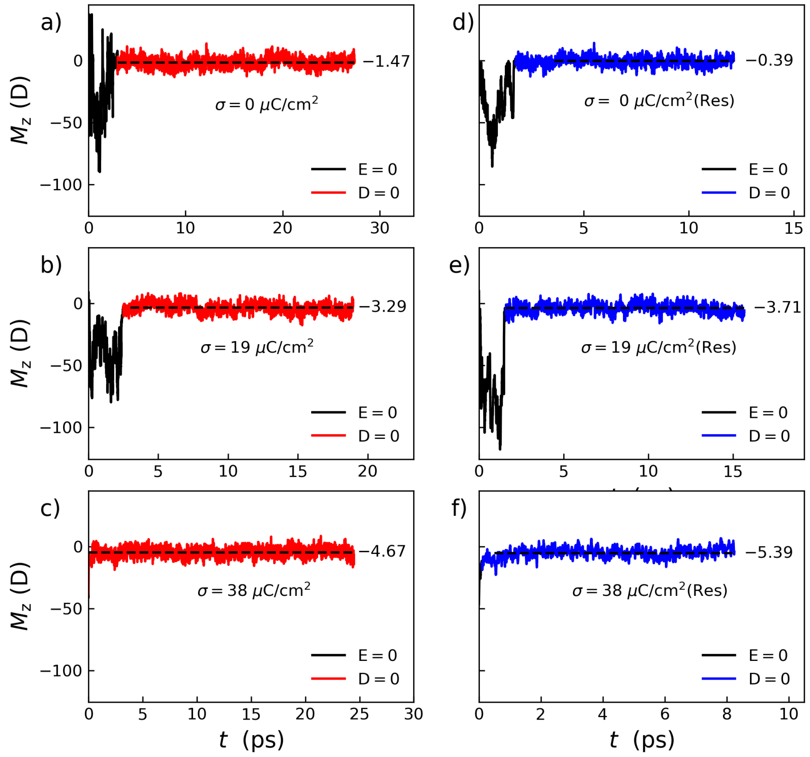


Figure S4: **Time evolution of total dipole moment $M_z$ at different surface charge densities.** The time evolution of total dipole moment $M_z$ for unrestrained (a, b and c) and restrained (d, e and f) $SnO_2$(110)/NaCl interfaces at $\sigma = 0$, 19 and 38 $\mu C/cm^2$, when switching electric boundary condition from $\bar{E}=0$ (black line) to $\bar{D} = 0$. Black dashed line is the time average of $M_z$ in each case.

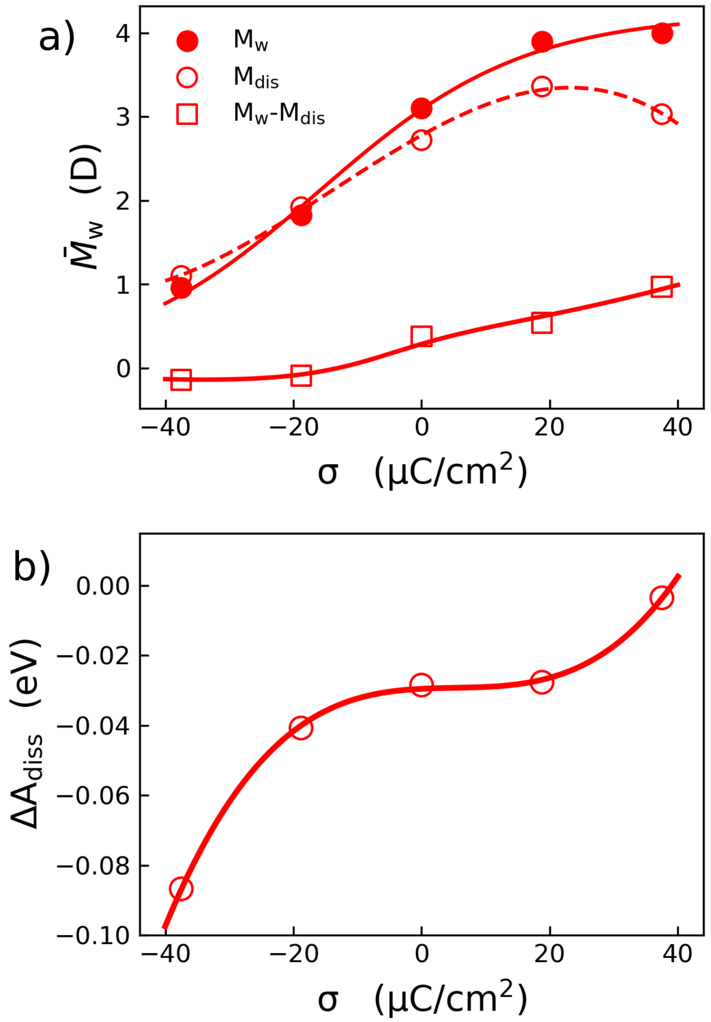


Figure S5: **Dipole moment and the free energy $\Delta A_{diss}$ of water dissociation.** (a) The average dipole moment of the adsorbed water molecules $M_w$, the dissociated ($OH^-$+$H^+$) $M_{dis}$, and the dipole moment changes of water dissociation $M_w - M_{dis}$. (b) Plot of the free energy of water dissociation $\Delta A_{diss}$ as a function of surfae charge density $\sigma$.

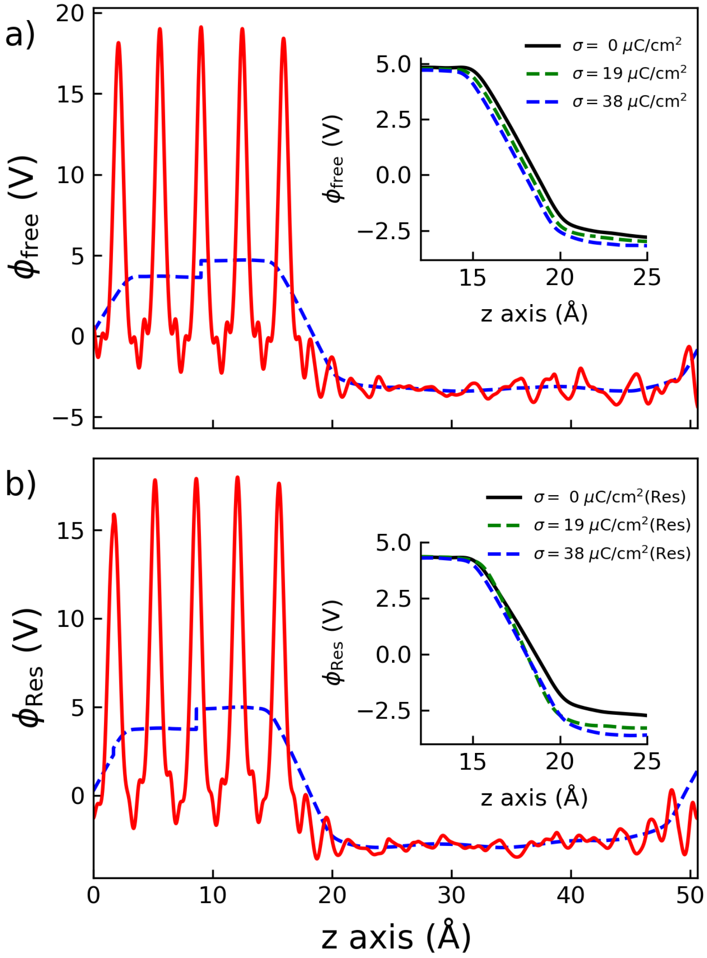


Figure S6: **Double layer potential at different charge densities.** Planar averaged (red solid line) and macro-averaged (dashed line) electrostatic potential $\phi$ for unrestrained (a) and restrained (b) $SnO_2$(110)/NaCl interfaces at $\sigma$=38 $\mu$C/cm$^2$. Inset: Macro-averaged electrostatic potential for the protonic side at $\sigma$=0, 19 and 38 $\mu$C/cm$^2$.

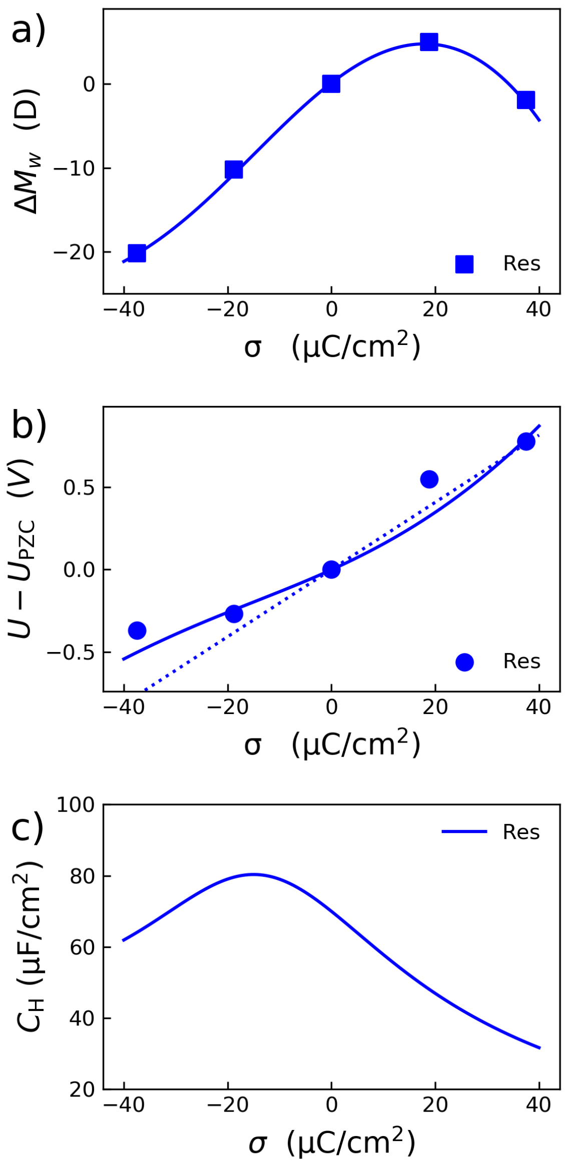


Figure S7: **Helmholtz capacitance of restrained $SnO_2$(110)/NaCl interface.** Plots of the total dipole moment changes $\Delta M_w$ (a), the electrostatic potential shift (b) and differential capacitance of the Helmholtz layer (c) versus surface charge density $\sigma$ for the restrained $SnO_2(110)/NaCl$ interface.

# References


(1) Zhang, C. Communication: Computing the Helmholtz capacitance of charged insulator-electrolyte interfaces from the supercell polarization. *J. Chem. Phys.* **2018**, *149*, 031103.

(2) Sayer, T.; Sprik, M.; Zhang, C. Finite electric displacement simulations of polar ionic solid-electrolyte interfaces: Application to NaCl(111)/aqueous NaCl solution. *J. Chem. Phys.* **2019**, *150*, 041716.

(3) Stengel, M.; Spaldin, N. A.; Vanderbilt, D. Electric displacement as the fundamental variable in electronic-structure calculations. *Nat. Phys.* **2009**, *5*, 304–308.

(4) Zhang, C.; Hutter, J.; Sprik, M. Coupling of Surface Chemistry and Electric Double Layer at $TiO_2$ Electrochemical Interfaces. *J. Phys. Chem. Lett.* **2019**, *10*, 3871–3876.

(5) Zhang, C.; Sprik, M. Finite field methods for the supercell modeling of charged insulator / electrolyte interfaces. *Phys. Rev. B* **2016**, *94*, 245309.

(6) Perdew, J. P.; Burke, K.; Ernzerhof, M. Generalized Gradient Approximation Made Simple. *Phys. Rev. Lett.* **1996**, *77*, 3865–3868.

(7) Hartwigsen, C.; Goedecker, S.; Hutter, J. Relativistic separable dual-space Gaussian pseudopotentials from H to Rn. *Phys. Rev. B* **1998**, *58*, 3641–3662.

(8) Goedecker, S.; Teter, M.; Hutter, J. Separable dual-space Gaussian pseudopotentials. *Phys. Rev. B* **1996**, *54*, 1703–1710.

(9) VandeVondele, J.; Hutter, J. Gaussian basis sets for accurate calculations on molecular systems in gas and condensed phases. *J. Chem. Phys.* **2007**, *127*, 114105.

(10) VandeVondele, J.; Mohamed, F.; Krack, M.; Hutter, J.; Sprik, M.; Parrinello, M. The influence of temperature and density functional models in ab initio molecular dynamics simulation of liquid water. *J. Chem. Phys.* **2005**, *122*, 014515.

(11) Hutter, J.; Iannuzzi, M.; Schiffmann, F.; VandeVondele, J. CP2K: atomistic simulations of condensed matter systems. *Wiley Interdiscip. Rev.: Comput. Mol. Sci.* **2014**, *4*, 15–25.

(12) VandeVondele, J.; Krack, M.; Mohamed, F.; Parrinello, M.; Chassaing, T.; Hutter, J. Quickstep: Fast and accurate density functional calculations using a mixed Gaussian and plane waves approach. *Comput. Phys. Commun.* **2005**, *167*, 103–128.

(13) Sulpizi, M.; Sprik, M. Acidity constants from vertical energy gaps: Density functional theory based molecular dynamics implementation. *Phys. Chem. Chem. Phys.* **2008**, *10*, 5238–5249.
